# Magnetic resonance evaluation of rotator cuff healing after surgical repair of large and massive lesions using the load-sharing rip-stop construct: encouraging results

**DOI:** 10.1016/j.jseint.2025.02.020

**Published:** 2025-03-27

**Authors:** Raul Meyer Kautsky, Benno Ejnisman, Juarez Leite Junior, Eduardo Antônio de Figueiredo, Ricardo Azeredo Costa, Jair Simmer Filho

**Affiliations:** a Hospital Santa Rita–Vitória, Brazil; b Universidade Federal de São Paulo (Unifesp), São Paulo, SP, Brazil; c Unimed Diagnóstico, Multiscan e Santi Vitória, Brazil; d Hospital Santa Rita - Vitória/ES and Hospital Estadual Dr. Jayme Santos Neves–Vitória, Brazil

**Keywords:** Shoulder joint, Arthroscopy, Rotator cuff injuries, Shoulder injuries, Double-row suture bridge, Arthroscopic rotator cuff repair, Radiographic outcomes, Clinical outcomes

## Abstract

**Background:**

The purpose of this study is to evaluate the tendon healing rate in large and massive tears using magnetic resonance imaging (MRI) based on the Sugaya classification and to present the functional outcomes (American Shoulder and Elbow Surgeons, University of California, Los Angeles, and visual analog scale scores) in patients who underwent the Load-Sharing Rip-Stop (LSRS) suture technique. These patients often present with retracted lesions, limited mobility, poor-quality tissues prone to tearing, medial tears, and/or insufficient bone quality (osteopenia or osteoporosis).

**Methods:**

Retrospective evaluation of patients aged > 55 years who underwent arthroscopic rotator cuff repair due to retracted tears (that after appropriate releases, it was not possible to cover more than 50% of the tendon footprint on the greater tuberosity upon rotator cuff traction), operated between 2014 and 2022, using the LSRS technique, and who underwent a minimum follow-up of 1 year, including postoperative MRI assessment.

**Results:**

Eighteen shoulders were included in the study and all of them performed a postoperative MRI; 1 patient experienced tendon rerupture on follow-up MRI, corresponding to a 5.56% failure rate. In the functional analysis, 16 shoulders (88.89%) scored above 80 on the American Shoulder and Elbow Surgeons score and had satisfactory results on the University of California, Los Angeles score (rated as excellent and good).

**Conclusion:**

In an average follow-up of 28 months, the LSRS suture technique demonstrated satisfactory functional outcomes and a good healing rate in patients with retracted and low-mobility rotator cuff injuries. These findings reinforce the idea that the LSRS technique can be a valuable option in managing these challenging cases.

Repairing the rotator cuff can be challenging in certain situations, especially when there’s a medial tendon tear, retraction, or limited tendon mobility even after adequate releases, when dealing with poor-quality tissues that offer an increasing risk of suture cutting through tendon, and when bone tissue is insufficient (osteopenia/osteoporosis). These scenarios are frequently encountered in chronic and retracted tears or when there’s tissue loss and severe tendinopathy, particularly in elderly patients.^41^ In such situations, conventional rotator cuff repair techniques become challenging.

Several factors can influence rotator cuff healing, including patient characteristics (osteoporosis, age, and smoking), tears characteristics (size, presence of tendon retraction, tendinopathy, muscle atrophy, and fatty infiltration),^6^^,^^14^^,^^27^^,^^28^^,^^34^ and intervention characteristics (surgical technique: tendon tension after repair, area/contact pressure, and biomechanical factors such as repair strength; suture tension which may vary depending on the surgical technique employed; and rehabilitation protocol used).^23^^,^^37^^,^^38^

Rotator cuff repair techniques continue to evolve, yet there remains significant debate over the optimal approach, with no universally accepted gold standard.^5^ The double-row suture bridge (DRSB) technique is gaining popularity due to its biomechanical advantages and healing rates.^10^^,^^25^ However, its application can be challenging in cases of retracted tendons with limited mobility, where this type of suture becomes difficult to execute. Denard & Burkhart described a unique surgical technique that is particularly valuable in these challenging situations. Load-Sharing Rip-Stop (LSRS) offers sutures specifically designed to better resist tendon suture cut-through, while also providing load distribution across 2 rows of independent yet supportive anchors. This provides greater tissue tear resistance and reduces mechanical stress on bone tissue.^8^^,^^9^ This technique was innovative because it provided a new treatment option for lesions with limited mobility, especially when covering more than 50% of the tendon footprint on the greater tuberosity is not possible. This refers to the dimension extending from the medial to the lateral aspect, meaning the tendon cannot be fully lateralized to its anatomic insertion, using anchors arranged in 2 independent rows where the suture threads intertwine, creating a “rip-stop” effect. This increases the likelihood of success and helps prevent tissue failure. The LSRS technique may be particularly useful in treating large and massive tears, serving as an alternative for those not amenable to double-row repair.^32^^,^^33^ However, there are limited research on the use of this technique.

In this study, we report clinical and imaging outcomes in patients with medial or retracted lesions with limited mobility who underwent LSRS technique. To evaluate tendon healing quality using magnetic resonance imaging (MRI) according to Sugaya classification^42^ and to present functional outcomes according to American Shoulder and Elbow Surgeons (ASES),^19^ University of California, Los Angeles (UCLA),^35^ and visual analog scale (VAS) for pain.^17^^,^^26^ The objective is to provide a descriptive analysis of healing rates in this unique cohort with this specific type of injury and discuss how they relate to previously reported outcomes of conventional techniques.

## Materials and methods

The study was conducted between January 2022 and February 2024. Institutional review board approval was obtained before the study commenced. Patients were selected from the last author (J. S. F.) private clinic database (electronic medical record, using the keyword “load-sharing rip-stop”), diagnosed with rotator cuff injury, and operated using the LSRS technique between January 2014 and July 2022. This was a retrospective, observational, descriptive, and analytical study analyzing image and clinical data of a group of patients who underwent repair of retracted posterosuperior rotator cuff lesions using the LSRS technique.

Inclusion criteria were agreement and signing of informed consent form, arthroscopic surgical repair of rotator cuff injury using LSRS technique, age more than 55 years, intraoperative findings of medial tendon injuries: tears with limited mobility after adequate releases, covering an area smaller than or equal to 50% of the original insertion in the greater tuberosity—in cases where the tendon cannot be fully lateralized to its anatomic insertion, intact subscapular tendon or suitable for surgical repair, large or massive lesions according to Cofield classification (> 3 cm in maximum dimension), involvement of 2 or more tendons, revision surgeries, and perform a postoperative MRI at least 1 year after surgery.

Exclusion criteria were partial posterosuperior repair (incomplete coverage of the superior portion in the humeral head footprint), irreparable subscapular lesion, preoperative presence of arthritis (Hamada classification ≥ 3), noncompliance with rehabilitation protocol, and loss to follow-up.

Patients who underwent rotator cuff repair are included in a research database maintained by the investigators. To identify potential candidates for this study, we searched the database using the keywords “rotator cuff” and “Load-Sharing Rip-Stop.” From the identified patients, those who agreed to participate underwent a medical consultation with surgeons (first and last authors) for clinical evaluation according to the research protocol. A detailed medical history was obtained; physical examination focused on the shoulder was performed; and ASES,^19^ UCLA,^35^ and VAS^17^^,^^26^ scores were recorded. Patients were requested to provide preoperative imaging examinations. MRI was chosen to evaluate tendon healing at a minimum follow-up of 1 year postoperatively. Preoperative lesion characteristics were assessed using the Patte classification^40^ to evaluate retraction and the Cofield^11^ classification to determine tear size. Sugaya classification was used to evaluate tendon healing^42^ and interpreted according to Muniandy et al’s criteria.^30^ Goutallier classification^14^ and the Tangent sign^44^ were used to grade muscle fatty infiltration. Imaging was evaluated by an experienced radiologist trained in musculoskeletal imaging.

### Surgical technique description

Surgery was performed in the beach chair position with the arm supported by the first assistant. In addition to the traditional posterior, anterior, and lateral portals, the posterolateral portal with 70° angulation optics was routinely used for bursal visualization. Advanced mobilization techniques described by Burkhart^21^^,^^22^ were applied in all cases to ensure adequate tendon mobilization before repair. When necessary, appropriate releases of the rotator interval (anterior and/or posterior) were performed. Additionally, margin convergence repair (side-by-side sutures with stitches at the tendon-tendon interface) was occasionally used as an adjunct technique.

**Figure 1:**
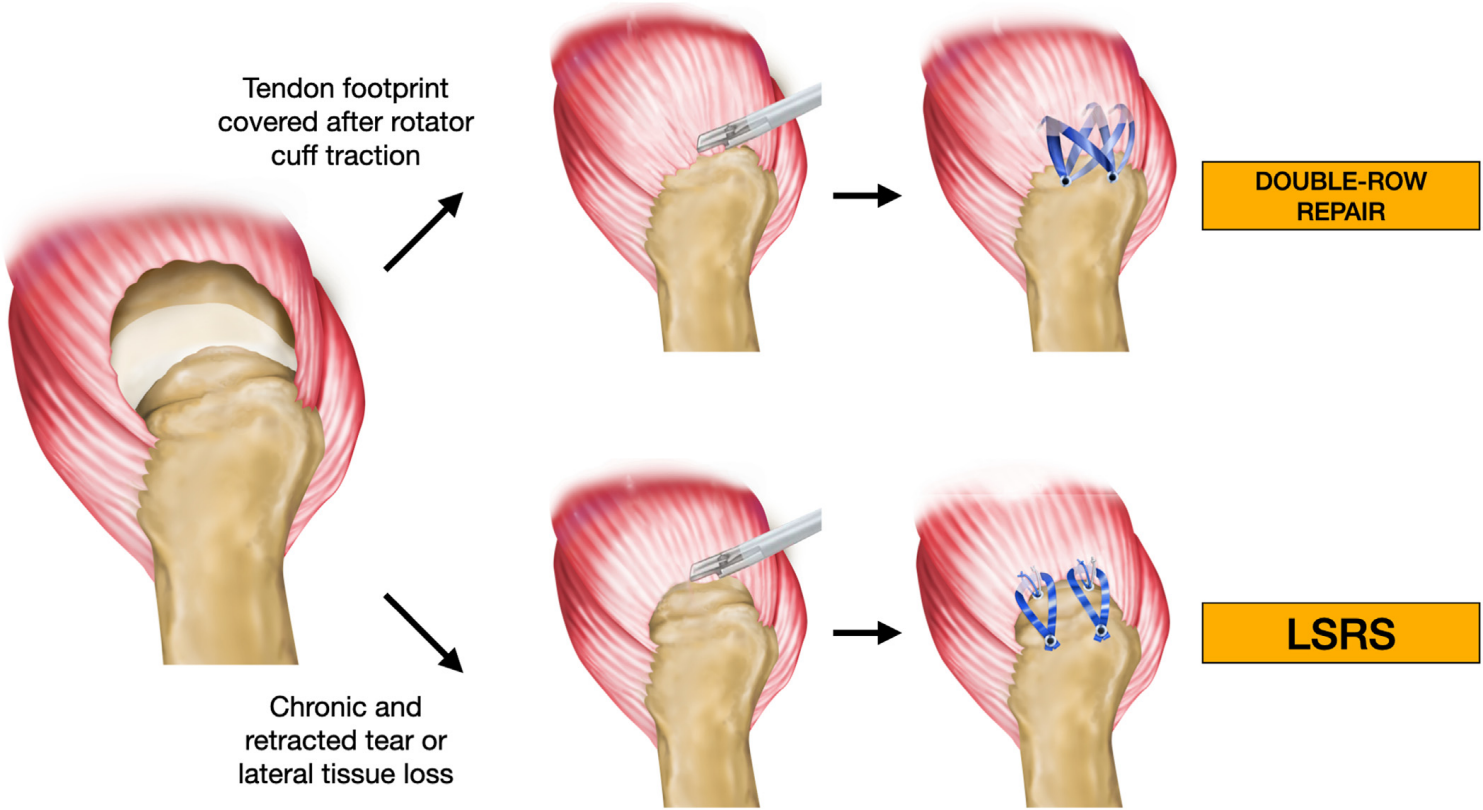
Initial image showing a rotator cuff tear that, depending on tissue mobility, may be treated differently. When after carrying out the necessary releases, and pulling the cuff laterally, we may face 2 scenarios: if there is a good mobility, we can perform a standard repair as the double row technique; but, if it is not possible to cover more than 50% of the tendon footprint in the greater tuberosity, the LSRS is an option. *LSRS*, Load-Sharing Rip-Stop.

We use the LSRS technique in patients with large and massive lesions according to the Cofield classification,^11^ medial tears, and retracted tendons with little mobility during surgery, according to the criteria of Noyes et al.^32^ In other words, when after carrying out the necessary releases, and pulling the cuff laterally, it was not possible to cover more than 50% of the tendon footprint in the greater tuberosity. Thus, these injuries are not suitable to be repaired with the double-row technique (interconnected anchors). This scenario normally occurs when we are dealing with poor tissue quality tendons (susceptible to tearing), in ruptures that occur medially (in the myotendinous junction and there is a remaining lateral stump connected to the footprint), when there is a lateral tendon loss or in cases with low tendon mobility (greater tension)^16^ (Fig. 1).

**Figure 2:**
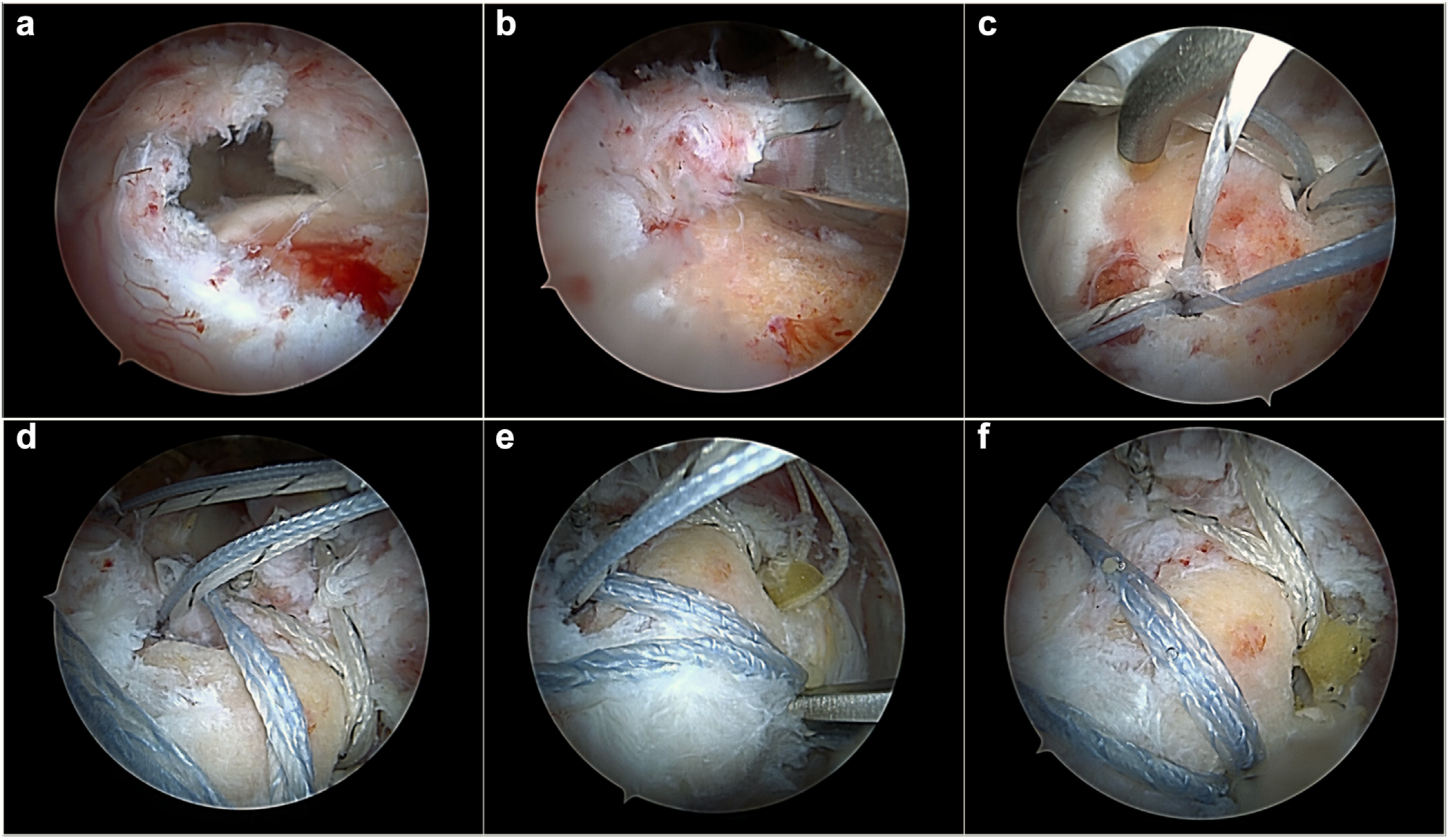
A right shoulder view in a patient being operated at the beach chair position through the posterolateral portal using a 70° scope. (**A**) “U”-shaped rotator cuff tear. (**B**) After carrying out the necessary releases, and pulling the cuff laterally with grasper, it was not possible to cover more than 50% of the tendon footprint in the greater tuberosity. (**C**) Two double-loaded anchors are placed on the great tuberosity at the limit point of tendon lateralization. (**D** and **E**) The rip-stop suture tapes are retrieved and fixed to the lateral cortex of the humerus with 2 knotless suture anchors. (**F**) The simple sliding knots (Samsung Medical Center sliding knot) of the double-loaded anchors are tightened using the knot pusher.

The development of the LSRS construct was previously described in detail by Denard & Burkhart.^9^^,^^32^ A 2-mm suture tape (FiberTape; Arthrex, Naples, FL, USA) is passed through the rotator cuff, with a transverse stitch in an inverted mattress configuration, passing 1 cm medial to the free edge of the tendon, not exceeding the medial limit of 3 mm lateral to the myotendinous junction (in cases where there is partial loss of tendon tissue). Next, a double-loaded anchor (BioComposite Corkscrew FT; Arthrex, Naples, FL, USA) is placed on the great tuberosity at the limit point of tendon lateralization during traction tests performed by the surgeon with grasper while the arm is holding in a position of neutral rotation and with 0°-30° of abduction. The suture threads of this anchor are used to create simple stitches, passed through the tendon medially to the suture tape, thus forming a rip-stop configuration, preventing tissue tearing tissue. After this, the rip-stop suture tapes are retrieved and fixed to the lateral cortex of the humerus with 1 knotless suture anchor (Knotless BioComposite SwiveLock C; Arthrex, Naples, FL, USA). At this point in the surgery, it is important to emphasize that to obtain the desired rip-stop mechanical effect, the surgeon must make sure that the suture threads of the double-loaded anchor are kept within the triangle formed by the tape. Another important detail is that the tape fixation tension should not be excessive, but only try to bring the lateral edge of the tendon over the double-loaded anchor previously positioned on the greater tubercle. Finally, the simple sliding knots (Samsung Medical Center sliding knot), backed up by 3 stacked knots with reverse hitches and alternating posts, of the double-loaded anchor are tightened using the knot pusher. If necessary, according to the size of the lesion, mobility, and quality of the tissue, another LSRS fixation system can be mounted alongside, following the same technique. In particular, we include a second LSRS system when the tear is too large in the anteroposterior direction (Fig. 2). In some situations, other double-loaded anchors were used to fix the edges of the lesions with modified Mason-Allen stitches. In these cases, these sutures are interconnected to the anchor in the lateral cortex with the intention of compressing and stabilizing the edges of the lesion against the bone, thus increasing the contact pressure and the contact area of the footprint.

We perform associated procedures such as acromioplasty, tenotomy of the long head of the biceps (with or without tenodesis), and distal clavicle resection, according to previous symptoms and intraoperative findings.

Postoperatively, we maintained an abduction sling for 6 weeks and recommended basic hand, wrist, and elbow exercises in the immediate postoperative period. After 6 weeks, we removed the sling and started physical therapy to gain active-assisted mobility in all directions. At 3 and a half months postoperatively, we started strengthening, assisted by a physiotherapy professional.

### Statistical analysis

A descriptive analysis was carried out for the collected data. Due to the small sample size (n = 18), confidence intervals (CIs) for proportions were calculated using the exact method. To calculate the CIs for the mean, the adherence of the probability distribution of each variable to the normal distribution was first tested using the Ryan-Joiner test, only being calculated the CIs for the mean of the variables for which there was such adherence.^29^

All CIs were calculated with 95% confidence and all hypothesis tests were carried out adopting a significance level of 5%, thus rejecting hypotheses with a descriptive level (*P* value) lower than 5%.

Statistical analyses were carried out by a statistician using Minitab Statistical Software v.21 (Minitab LLC, State College, PA, USA) under the supervision of the research team.

## Results

**Figure 3:**
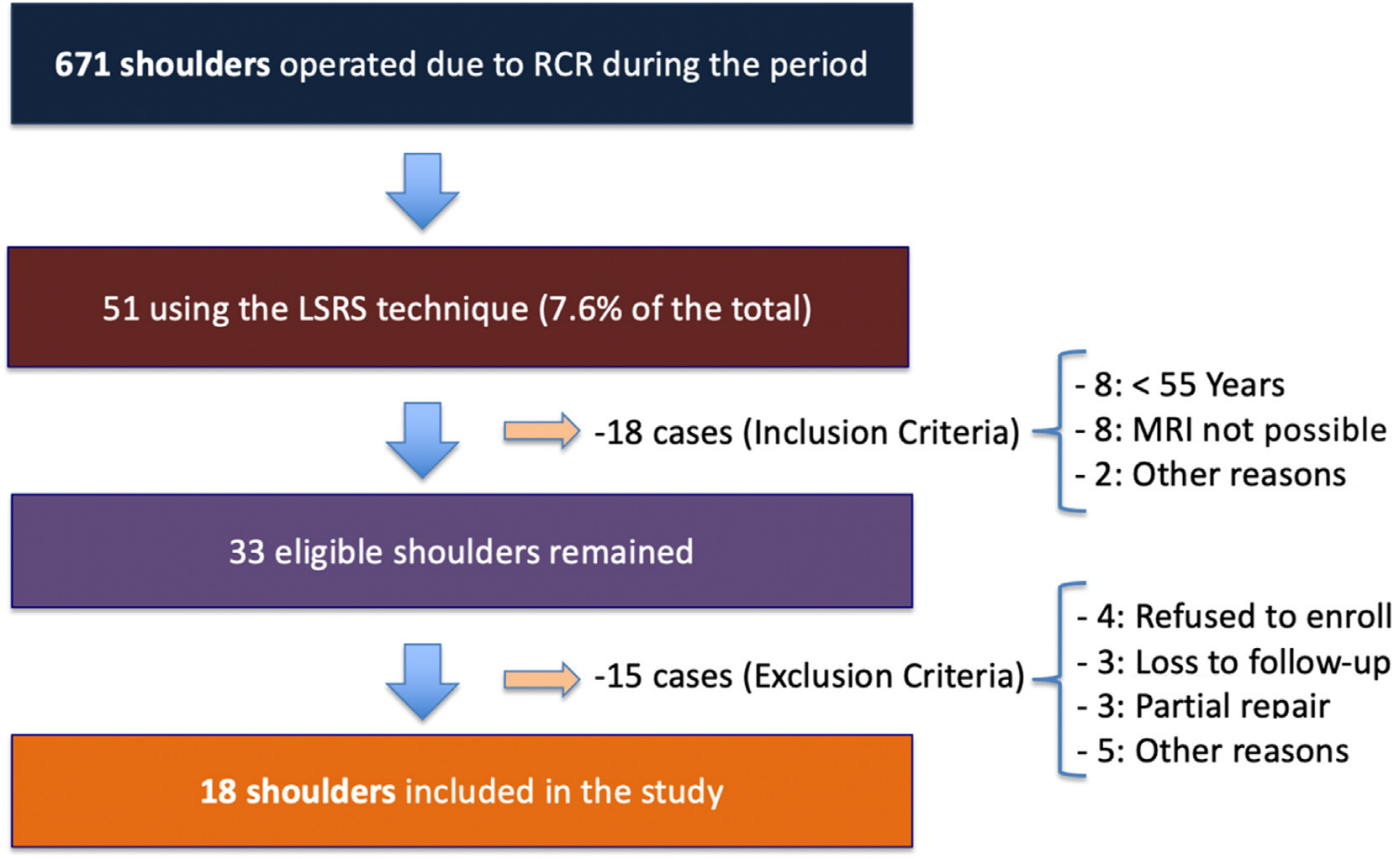
Flowchart illustrating the selection process of operated shoulders included in the study. The total number of shoulders operated due to rotator cuff tears (RCTs) was 671. After applying the inclusion and exclusion criteria, 18 shoulders remained eligible for the study. *MRI*, magnetic resonance imaging; *LSRS*, Load-Sharing Rip-Stop; *RCR*, rotator cuff repair.

As shown in the flowchart of this study (Fig. 3), 18 shoulders (17 patients) were selected and underwent a complete evaluation, including clinical and imaging analysis. This final group was included in the full statistical analysis. It is important to note that 8 other patients, outside the final study group, chose not to undergo follow-up imaging and were therefore not included in this study. However, in the clinical evaluation of these patients, 7 achieved satisfactory outcomes on the UCLA score (4 excellent, 3 good, and 1 fair).

In the end, 17 patients (18 shoulders) responded to the call to participate in the research and carried out all stages of the work. All had undergone surgery to treat rotator cuff lesions as the main diagnosis. Regarding gender, 10 cases (55.6%) were females and 8 (44.4%) were males. The average age was 65 years (range: 56-75 years). Regarding age distribution, 55.6% of patients were aged less than 65 years, while 44.4% were older. The average period of follow-up and postoperative MRI was 28 months, with a range of 12-70 months. The follow-up and MRI were performed within up to 1 month of each other, but not during the same visit. The right shoulder was affected in the majority of cases (83.3%), and the dominant shoulder was affected in 77.8% of cases (Table I).

**Table I:** Demographic data (N = 18 shoulders).

	Average	Standard deviation
Age (yr; range)	65.1 (56-75)	6.4
Follow-up (mo; range)	28.4 (12-70)	18.9

	N absolutes	Percentage (%)

Sex	10 ♀; 8 ♂	55.6% ♀; 44.4% ♂
Laterality	15 right; 3 left	83.3% right and 16.7% left
Dominance	14 yes; 4 no	77.8% dominant

*N*, numbers; ♀, female; ♂, male.

All cases involved large or massive tears according to Cofield’s classification.^11^ Since surgical videos were available for all procedures, we also confirmed that these were retracted or low-mobility lesions according to the criteria of Noyes et al.^32^ Among the 18 cases, 2 were revision surgeries.

**Figure 4:**
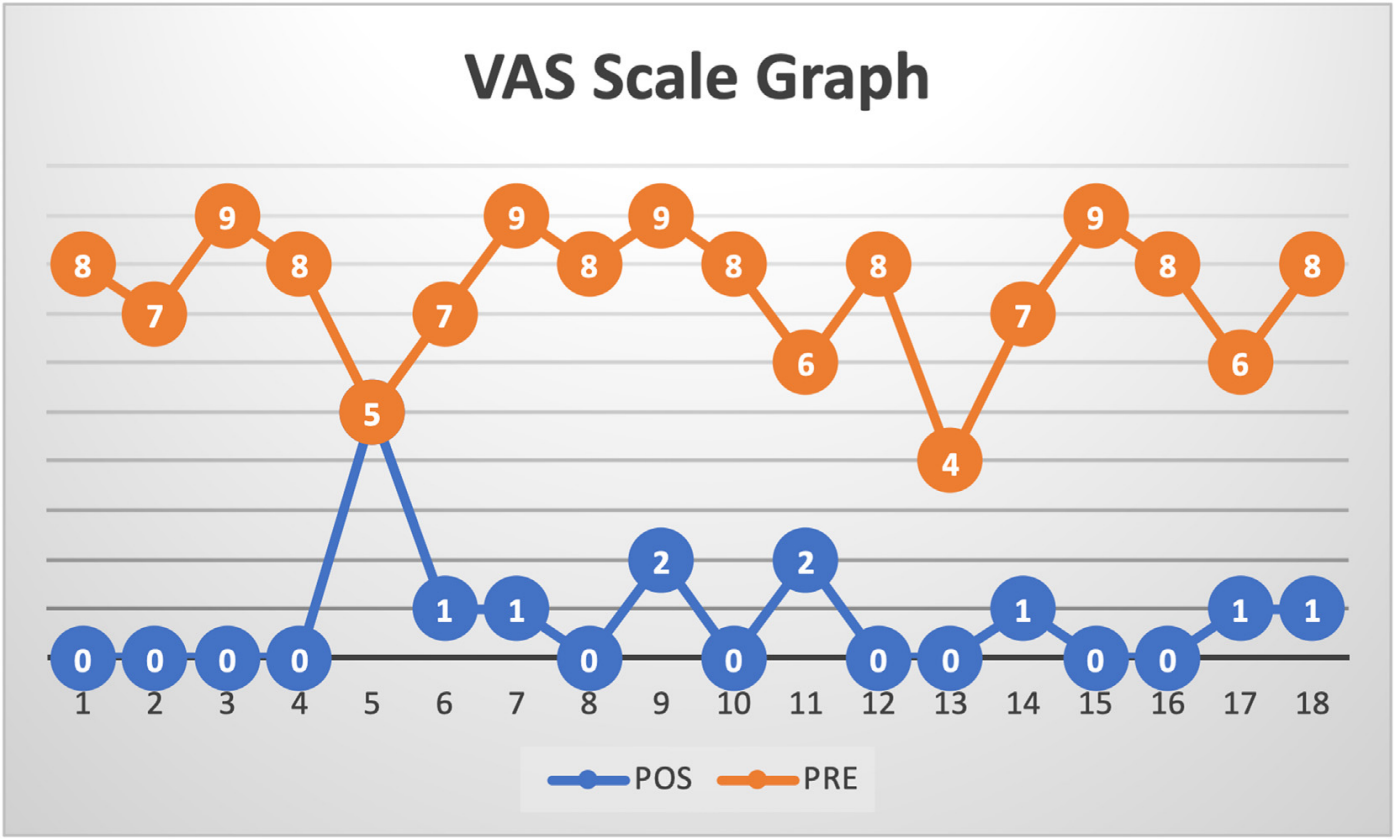
Graph showing the score on the VAS scale in the preoperative and postoperative periods. *Red line*: situation before operating; *Blue line*: situation after surgery. *VAS*, visual analog scale.

Pain intensity was reduced in a statistically significant way after surgical intervention (*P* < .001). Before surgery, participants reported an average of 7 on the VAS, while at final evaluation this average decreased to 1 (graph 1–VAS). This difference observed between preoperative and postoperative means suggests the effectiveness of the surgical procedure in relieving pain related to the condition of the rotator cuff (Fig. 4).

The comorbidities presented by the patients are summarized in Table II. It is worth mentioning that some patients presented more than 1 comorbidity.

**Table II:** Comorbidities.

	Absolute number	Percentage
Dyslipidemia	11	61.1%
Arterial hypertension	7	38.9%
Diabetes melitus	2	11.1%
Hypothyroidism	2	11.1%
Smoking	1	5.6%
Rheumatologic disease	1	5.6%
Working compensation	1	5.6%

Table illustrates the comorbidities observed in the study population, presented as absolute numbers and percentages.

Functional analysis at final follow-up, measured by ASES and UCLA scores, demonstrated positive results (Table III). The average ASES score was 91, with a range of 68 to 100 and a standard deviation of 9.2. The UCLA score had a mean of 31, ranging from 22-35, with a standard deviation of 3.6. Stratifying the UCLA score, 33.3% of patients obtained excellent results (34-35 points), 55.6% good (28-33 points), and 11.1% fair (21-27 points), with no records of poor results (0-20). Thus, 88.9% of the shoulders were considered satisfactory and only 11.1% unsatisfactory. Likewise, stratifying the ASES score, 88.9% of shoulders had scores above 80 points and 11.1% below 80 points (Table III).

**Table III:** Stratification of postoperative functional scores (N = 18).

Score	Stratification	Shoulders	Percentage/interpretation	
ASES	> 80 points	16	88.9%	
	< 80 points	2	11.1%	
	Average = 91 points		Standard deviation = 9.2	
UCLA	Excellent	6	33.3%	UCLA
	Good	10	55.6%	
	Reasonable	2	11.1%	
	Bad	0	0%	
	Average = 31.4 points		Standard deviation = 3.6	
VAS	Preoperative average = 7		Postoperative average = 1∗	

*ASES*, American Shoulder and Elbow Surgeons; *UCLA*, University of California, Los Angeles; *VAS*, visual analog scale.

ASES and UCLA scores; N = number of shoulders assessed.

∗ At a significance level of 5%, it was concluded that the mean VAS score is lower in the postoperative period (*P* = .000).

Injuries were categorized as degenerative (72.2%), acute chronic injury–traumatic (16.7%), and reruptures after previous surgery (11.1%) (Table IV).

**Table IV:** Categorization of tear patterns in the study population.

	Absolute number	Percentage
Degenerative	13	72.2%
Acute chronic injury–traumatic	3	16.7%
Revision surgery	2	11.1%

Categorization of tear patterns in the study population, illustrating the different underlying conditions that led to surgical treatment.

Preoperative MRI was available in 12 of the 18 operated shoulders. In these cases, fatty infiltration of the supraspinatus was classified according to Goutallier as 0 = 1 case (8.3%), 1 = 4 cases (33.3%), 2 = 3 cases (25%), 3 = 3 cases (25%), and 4 = 1 case (8.3%). The Tangent sign was considered positive in 4 cases (25%). Five patients were classified as Patte 1 (41.7%), 5 patients as 2 (41.7%), and 2 patients as 3 (16.7%) (Table V).

**Table V:** Analysis of magnetic resonance imaging.

Preoperative status (N = 12)		
Goutallier classification (supraspinatus muscle)		
Grade 0	1 (8.3%)	8 (66.7%)–mild or moderate∗
Grade 1	4 (33.3%)	
Grade 2	3 (25%)	
Grade 3	3 (25%)	4 (33.3%)–advanced∗
Grade 4	1 (8.3%)	
Patte’s classification		
Type 1	4	33.3%
Type 2	6	50%
Type 3	2	16.7%
Tangent sign		
Negative	8	66.7%
Positive	4	33.3%

Postoperative situation (N = 18)		
Goutallier classification (supraspinatus muscle)		
Grade 0	1 (5.6%)	38.9%–mild or moderate∗
Grade 1	3 (16.7%)	
Grade 2	3 (16.7%)	
Grade 3	5 (27.8%)	61.1%–advanced∗
Grade 4	6 (33.3%)	
Sugaya classification (evaluate disruption)		
Grade I	0	17 (94.4%)–healing
Grade II	7	
Grade III	10	
Grade IV	1	1 (5.6%)–rerupture
Grade V	0	
Comparison of Goutallier (preoperative vs. postoperative)		
No change	9	75%
Worsening	3	25%

Classification of lesions in the preoperative and postoperative periods. N = number of shoulders assessed.

∗ Interpretation of fat degeneration in Goutallier’s classification; Grades 0, 1, and 2 are considered to be mild and 3 and 4 advanced.

Postoperative MRI was the method of choice to evaluate tendon healing using the Sugaya classification as a reference for its interpretation,^42^ according to the criteria of Muniandy. et al.^30^ In our sample (18 shoulders), no shoulder was classified as type I or V, 7 shoulders were considered type II (38.9%), 10 shoulders were considered type III (55.6%), and 1 shoulder was considered type IV (5.6%). Thus, the interpretation was that 17 shoulders (94.4%) showed satisfactory healing and only 1 case (5.6%) showed failure to heal/rerupture (Table V).

When we compared the Goutallier classification for the supraspinatus tendon (main tendon affected) between patients who had MRI scans available before and after surgery, we found that in 9 cases (75%), there was no change in classification; while in 3 shoulders (25%), there was a worsening of the Goutalier classification (Table V). Despite this, there was no statistical relationship between imaging examinations and postoperative functional assessment; in fact, even patients who had worsening of the Goutallier classification presented satisfactory functional results after surgery.

The details of the surgical procedure are summarized in Table VI. Acromioplasty was performed in 77.8% of cases; in 66.7%, it was necessary to perform anterior and/or posterior interval releases; in 46.7% of cases, resection of the distal portion of the clavicle was performed; the presence of delamination as described by Boileau et al^3^ was found in 27.8%. Regarding other findings: chondral lesions were identified in 22.2%; os acromiale was observed in 11.1%, with 1 case being a preacromion and the other a meso-acromion—both were resected during surgery; and in 1 shoulder (5.6%), the injury was more medial (at the myotendinous transition; and there was a remaining tendon stump distal to the rupture).

**Table VI:** Surgery details.

	N	Percentage
Cuff repair		
Conventional total	17	94.4%
Total myotendinous transition	1	5.6%
Long head of the biceps tendon		
Previously broken	2	11.1%
Tenotomy	11	61.1%
Tenodesis	5	27.8%
Subscapularis (type of lesion)		
No lesion found	5	27.8%
1/3 superior∗	7	38.9%
Complete	6	33.3%
Subscapularis (approach)		
No specific action taken	5	27.8%
Tendon-tendon stitch	1	5.6%
Single row	9	50.0%
Double row	3	16.7%
Acromioplasty		
No	4	22.2%
Yes	14	77.8%
Delamination (Boileau criteria)		
No	13	72.2%
Yes	5	27.8%
Distal clavicle excision		
No	10	55.6%
Yes	8	44.4%
Interval release		
No	6	33.3%
Yes	12	66.7%
Anchors used		
Total (average/change)	4	(2-7)
Number of patients who used 1 or 2 knotless anchors from the total		
One knotless anchor (simple LSRS)	10	55.6%
Two knotless anchors (double LSRS)	8	44.4%

*LSRS*, Load-Sharing Rip-Stop.

∗ Lesion of the upper third of the subscapularis tendon.

The subscapularis tendon was intact in 5 cases (27.8%) and did not require a specific approach; in 7 cases (38.9%), there was a lesion in the upper third; and in 6 shoulders (33.3%), there was a complete lesion of the subscapularis tendon. Repairs of the subscapularis tendon included a tendon-to-tendon stitch in 1 patient (5.6%), single-row repair in 9 shoulders (50%), and double-row repair in 3 shoulders (16.7%).

The long head of the biceps was previously torn in 2 shoulders (11.1%); in 11 cases (61.1%), tenotomy was performed; and in 5 shoulders (27.8%), tenodesis of the long cable of the biceps was performed at the top of the bicipital groove (using the Biceps Swivelock system).

The average number of anchors used was 4 anchors per surgery (range: 2-7). In 10 shoulders (55.6%), 1 knotless anchor was used, and in 8 shoulders (44.4%), 2 knotless anchors were used.

## Discussion

The most important results of this work are the high rate of tendon healing (low rate of rerupture) after arthroscopic repair of the rotator cuff and clinical improvement (measured by functional scores) in a patient population that is traditionally difficult to treat. These types of lesions are often associated with poor tendon quality, and in elderly populations, osteopenia or osteoporosis is frequently observed. Both factors are known to increase the risk of rerupture or repair failure.^12^^,^^41^ Our findings are encouraging when compared with previous reports of repairs performed using the single-row technique for large and massive rotator cuff tears.^2^^,^^13^ In our study, 94.4% of patients achieved partial or complete healing of the rotator cuff in the posterosuperior portion of the humeral head, as determined by MRI.

In our case series, only 1 shoulder (5.6%) was classified as Sugaya IV on the control imaging examination and was interpreted as a case of failed healing/rerupture. In the literature, there is a discussion whether or not cases classified as Sugaya III should be considered a rerupture. In our study, we followed the validation by Muniandy. et al^30^ and used it as a reference for interpreting Sugaya’s classification. Therefore, when the Sugaya classification result was I, II, or III, the tendon was considered healed, whereas IV and V were classified as retear. Although our interpretation may be debatable, there is no consensus in the literature on whether Sugaya III should be classified as partially healed or as a rerupture.^15^^,^^24^^,^^30^^,^^31^ Furthermore, since the patients in this study who fell into this category demonstrated good functional outcomes at follow-up beyond 1 year, classifying them as healed seemed to be the most reasonable approach.

It is important to mention that, contrary to expectations, the patient who presented tendon rerupture in the postoperative segment was clinically satisfied and presented satisfactory results in the functional scores (ASES and UCLA). A similar situation was reported by Galatz et al who documented excellent clinical and functional results despite a high rate of rerupture.^13^ This finding underscores a potential discrepancy between the patient’s subjective clinical evaluation and the objective findings from imaging studies, a phenomenon also reported by Vecchini et al^43^ and Kim et al.^18^ That highlights an intriguing aspect of clinical outcomes in tendon repair: while functional scores such as ASES and UCLA provide valuable insights, they may have inherent limitations in fully capturing the patient’s subjective experience, especially in cases of tendon rerupture. It is also noteworthy that patients with imaging-confirmed healing failures can still experience significant initial clinical improvement, as supported by previous studies. However, due to the follow-up of this study, it was not possible to assess whether this clinical improvement was sustained in the long term. It is important to highlight that this study did not directly compare DRSB repair or arthroscopic débridement as alternative treatment strategies. Regarding the DRSB technique, its application is generally reserved for cases where complete footprint coverage can be achieved without excessive tension. However, patients undergoing surgery following the LSRS protocol are not candidates for DRSB repair, making direct comparison with these cases less applicable.

As for arthroscopic débridement, we recognize that studies in the literature have shown good early and mid-term functional outcomes. However, we believe that, in the long term, tendon repair—even if only partially healed—attempts to improve the biomechanics of the shoulder^1^ and tends to provide better functional outcomes for the joint compared to isolated débridement. While this study did not specifically address this comparison, we consider that the sustained clinical benefits over time favor repair whenever feasible.

Our findings have similarities with the study conducted by Noyes et al which demonstrated a healing rate of 82.3%, in the postoperative evaluation with ultrasound after 6 months of surgery.^32^ While in our series, using magnetic resonance images, we obtained healing in 94.4%. Although the studies present methodological differences in relation to the images used and the follow-up time, we believe that both rates are encouraging considering the challenge of treating large and massive rotator cuff tears.

We chose MRI as the imaging method of choice for evaluating rotator cuff tendon healing due to its high resolution, multiplanarity capacity, absence of ionizing radiation,^7^^,^^36^ and to reduce the risk of failure and examiner-dependent variations that are inherent to the US method. It is worth noting that the MRI images were evaluated by an experienced radiologist who has no relationship with the surgical team, trained in the musculoskeletal system and with 15 years of clinical practice.

This study detected a reduction in pain intensity after rotator cuff surgery with statistical significance (*P* < .001), as evidenced by the decrease in the mean VAS from 7 to 1. This difference is indicative of the effectiveness of the procedure in relieving pain associated with the condition of the rotator cuff in the vast majority of cases studied.

Functional analysis of the shoulders after rotator cuff surgery revealed satisfactory results in 88.9% of cases, as indicated by ASES (> 80 points) and UCLA (> 28 points) scores. However, it is interesting to report that in the 2 cases that were classified as having unsatisfactory functional results, the MRI revealed good tendon healing (both were classified as Sugaya 2), revealing a dissociation between the imaging tests and the clinical result. This fact highlights the complexity in assessing postoperative shoulder function, making it possible to consider that functional scores do not completely capture the patient’s subjective perception of their shoulder function, which can be influenced by a variety of factors, including individual expectations, pain tolerance, and quality of life.

Rotator cuff revision surgeries pose a significant challenge for surgeons, often involving retracted lesions and tissue loss. In our study, we included revision cases where the LSRS technique was applied to address tendon reruptures. Although Noyes et al in 2017 considered revision surgeries as an exclusion criterion in their case series,^32^ we believe that considering the rip-stop property, the LSRS technique can be especially valuable in cases of tendon rerupture where we often encounter retracted lesions (low mobility), loss of lateral tissue, and poor-quality tendons (with great susceptibility to tearing). This perspective aligns with Ladermann et al who highlighted that revision scenarios frequently involve deficient tissues, either in the tendon or bone (or both), necessitating techniques such as arthroscopic bone grafting, rip-stop sutures, and load-sharing suture anchor constructs.^20^ By providing enhanced resistance to propagation of tears and optimizing load distribution, the LSRS technique may address these challenges effectively, offering a reliable solution in the complex setting of rotator cuff revisions.

However, even if care is taken with the appropriate indication and attentive execution of the surgical technique, obtaining satisfactory results can be difficult. In our study, we observed that the 2 cases of revision surgery resulted in good clinical results, as assessed by VAS, UCLA, and ASES functional scores. However, regarding healing, the shoulders were classified as Sugaya 3 and 4. Therefore, we consider that healing was satisfactory in one case and there was rerupture in the other case despite both being clinically satisfied.

We were able to carry out an MRI analysis of Goutalier classification in 12 shoulders that had preoperative images available, and comparing with the postoperative MRI. We found that there was no change in classification in 9 cases (75%), while in 3 shoulders (25%) there was worsening. Despite this, there was no statistical relationship between imaging examinations and postoperative functional assessment or retear, since even in patients who developed worsening fatty infiltration, we found satisfactory functional results.

It is worth mentioning that in our statistical analysis, we did not observe significant differences when comparing unfavorable outcomes (defined by the presence of tendon rerupture or unsatisfactory clinical results) with the presence of advanced fatty infiltration. However, this lack of significance may be attributed to the small sample size of our study, which likely limited the statistical power to detect such associations. This represents a potential limitation of our investigation, as larger cohorts might reveal meaningful relationships that our study was underpowered to demonstrate.

Our sample is relatively small in number; however, it is worth highlighting that because it is a specific topic and there are few studies in the literature that deal with the subject, the number of patients evaluated is comparable to the few studies in the literature. Furthermore, this study was unprecedented in reporting the results of tendon healing using MRI in the studied population. Therefore, considering the scarcity of clinical work in the literature on the LSRS technique in complex and retracted injuries (considered a challenge in surgical treatment), especially with regard to the analysis of tendon healing using MRI, we understand that this work provides information valuable for the shoulder surgeon.

Another limitation of this study is the less than 2-year minimum clinical follow-up. However, recent evidence indicates that this standard may not always add significant clinical utility. Patel et al demonstrated that patient-reported outcomes and achievement of clinically significant outcomes at 1 year postoperatively are highly comparable to those observed at 2 years, with minimal differences that are unlikely to be clinically relevant.^39^ Additionally, as highlighted in the editorial commentary by Brinkman J. C., the traditionally accepted 2-year follow-up benchmark for rotator cuff repair outcomes may be an arbitrary standard, with 1-year follow-up providing sufficient clinical insight.^4^ These findings support the relevance and reliability of short-term outcomes in studies such as ours.

Limitations: there is no comparison group; the lack of paired preoperative data (score completion) to measure clinical improvement after surgery with greater precision; less than 2-year minimum clinical follow-up; and the recovery of preoperative magnetic resonance images in only 12 of the 18 shoulders included in the study.

## Conclusion

In an average follow-up of 28 months, the LSRS suture technique demonstrated satisfactory functional outcomes and a good healing rate in patients with retracted and low-mobility rotator cuff injuries. These findings reinforce the idea that the LSRS technique can be a valuable option in managing these challenging cases.

## Acknowledgments

This paper was edited for proper English language, grammar, punctuation, spelling, and overall style by one or more of the highly qualified native English-speaking editors at AJE.

## Disclaimers

Funding: No funding was disclosed by the authors.

Conflicts of interest: Jair Simmer Filho and Benno Ejnisman report a relationship with Arthrex Inc. that includes speaking and lecture fees. The other authors, their immediate families, and any research foundation with which they are affiliated have not received any financial payments or other benefits from any commercial entity related to the subject of this article.
